# Comparison of Different Polymeric Membranes in Direct Contact Membrane Distillation and Air Gap Membrane Distillation Configurations

**DOI:** 10.3390/membranes15030091

**Published:** 2025-03-13

**Authors:** Cristiane Raquel Sousa Mesquita, Abdul Orlando Cárdenas Gómez, Carolina Palma Naveira Cotta, Renato Machado Cotta

**Affiliations:** 1 Laboratory of Nano & Microfluidics and Microsystems-LabMEMS, Mechanical Engineering Department, POLI & COPPE/UFRJ, Federal University of Rio de Janeiro, 360 Av. Moniz de Aragão, CT-2–Cidade Universitária, Rio de Janeiro 21941-594, Brazil; crismesquita@coppe.ufrj.br (C.R.S.M.); abdulcardenas@ufrj.br (A.O.C.G.); 2 Laboratory of Sustainable Energies Technologies, LATES-GTM, Navy Research Institute, IPqM/CTMRJ, General Directorate of Nuclear and Technological Development, DGDNTM, Brazilian Navy, 02 R. Ipiru–Cacuia, Rio de Janeiro 21931-095, Brazil

**Keywords:** water desalination, air gap membrane distillation, direct contact membrane distillation, heat and mass transfer, hydrophobic porous membrane

## Abstract

Membrane distillation (MD) is an evolving thermal separation technique most frequently aimed at water desalination, compatible with low-grade heat sources such as waste heat from thermal engines, solar collectors, and high-concentration photovoltaic panels. This study presents a comprehensive theoretical–experimental evaluation of three commercial membranes of different materials (PE, PVDF, and PTFE), tested for two distinct MD modules—a Direct Contact Membrane Distillation (DCMD) module and an Air Gap Membrane Distillation (AGMD) module—analyzing the impact of key operational parameters on the performance of the individual membranes in each configuration. The results showed that increasing the feed saline concentration from 7 g/L to 70 g/L led to distillate flux reductions of 12.2% in the DCMD module and 42.9% in the AGMD one, averaged over the whole set of experiments. The increase in feed temperature from 65 °C to 85 °C resulted in distillate fluxes up to 2.36 times higher in the DCMD module and 2.70 times higher in the AGMD one. The PE-made membrane demonstrated the highest distillate fluxes, while the PVDF and PTFE membranes exhibited superior performance under high-salinity conditions in the AGMD module. Membranes with high contact angles, such as PTFE with 143.4°, performed better under high salinity conditions. Variations in operational parameters, such as flow rate and temperature, markedly affect the temperature and concentration polarization effects. The analyses underscored the necessity of a careful selection of membrane type for each distillation configuration by the specific characteristics of the process and its operational conditions. In addition to experimental findings, the proposed heat and mass transfer-reduced model showed good agreement with experimental data, with deviations within ±15%, effectively capturing the influence of operational parameters. Theoretical predictions showed good agreement with experimental data, confirming the model’s validity, which can be applied to optimization methodologies to improve the membrane distillation process.

## 1. Introduction

Membrane distillation (MD) is a developing thermal separation technique that utilizes a hydrophobic porous membrane to selectively permit the transport of water vapor from a heated feed solution to a cooler permeate side. The driving force in MD is the vapor pressure gradient across the hydrophobic membrane surfaces, influenced by temperature differences and other operational and structural parameters [1,2,3,4,5]. Recent advancements in superhydrophobic membranes have demonstrated potential for enhancing mass flux by mitigating temperature polarization effects, which otherwise reduce the vapor pressure gradient. Such improvements have been explored through the development of novel membranes and modifications to existing commercial ones [6,7,8,9].

In comparison with reverse osmosis (RO), which is one of the most frequently employed desalination technologies, MD offers some interesting advantages that warrant a niche of application for this hybrid thermal membrane separation process. These include high rejection rates for non-volatile solutes, operation under low pressures, and compatibility with low-grade waste heat or renewable energy sources. It is also a viable alternative for diverse applications including the removal of organic heavy metals and contaminants from wastewater [10,11]. Additionally, MD achieves near-total rejection of non-volatile solutes, making it particularly suitable for treating high-salinity brines, such as RO concentrate waste streams or integrating with multiple-effect distillation (MED) systems [12,13,14,15,16,17]. Membrane distillation still presents a higher specific thermal energy consumption than RO but a relatively low electrical consumption, which can make it most interesting for applications that use low-grade waste heat sources or solar thermal energy [18]. In contrast, reverse osmosis presents a minimum theoretical energy consumption, being commonly applied for large water desalination volumes [19]. In decentralized or off-grid scenarios, where the availability of electrical energy is limited, MD is again a viable alternative for distributed cogeneration when integrated with waste heat and renewable energy sources [20]. The present work is part of a wider initiative on the development of sustainable cogeneration islands for decentralized electricity, water, and fuel provision in remote off-grid regions and humanitarian bases or natural disaster sites [21,22].

In this context, to evaluate and compare the performance of different commercial hydrophobic membranes for MD, an experimental setup was developed for the characterization of a flat-plate Direct Contact Membrane Distillation (DCMD) module. This setup allows for controlled variation of operational parameters, including feed and permeate temperatures and flow rates. The DCMD configuration is characterized by direct contact between the hot and cold solutions (feed and distillate) with the membrane, facilitating vapor transport and condensation [23,24]. However, there are some alternative configurations that have received substantial attention in the literature, such as Air Gap Membrane Distillation (AGMD). The AGMD configuration incorporates an air gap between the membrane and the condensing surface, acting as a thermal barrier that reduces heat losses through the membrane and enhances thermal efficiency [23,25]. While DCMD is known for offering ease of operation and higher vapor fluxes, AGMD provides superior thermal efficiency, requiring a trade-off based on specific process requirements. Thus, a second experimental setup was assembled, again with full control of the operational variables, to allow for critical performance comparisons of different commercially available polymeric membranes, operating on either of the two configurations.

Although a direct comparison of the DCMD and AGMD configurations is not the aim of this work, which focuses on the comparison of commercial membranes of different materials installed in two different modules, it is worthwhile to briefly discuss previous contributions and niches of each configuration. DCMD excels in reverse osmosis tailing desalination where high flow rates are required, while AGMD is known for being more efficient in processes that are more energy demanding, such as zero liquid discharge (ZLD) systems [25]. Both technologies have been applied in the treatment of mining wastewater, recovering valuable metals such as copper and gold, and critical metals such as lithium [10,26,27,28]. In biotechnology, DCMD has been used in the concentration of biological solutions and separation of proteins, preserving heat-sensitive molecules [29,30,31,32]. The pharmaceutical industry employs AGMD to concentrate biomolecules, resulting in high permeate purity [4,33]. In the treatment of radioactive effluents, AGMD has been used to reduce the volume of waste and ensure the safe containment of radionuclides, while DCMD desalinates contaminated water near nuclear facilities, ensuring high-quality water treatment [34,35,36]. AGMD has been employed to remove hydrocarbons and salts in the oil industry while DCMD has been used to remove organic pollutants, enabling the recycling of water obtained from oil and gas extraction [37,38]. Both technologies also generate drinking water out of brackish or seawater, particularly in remote areas [39,40,41]. These characteristics reinforce the importance of identifying ideal scenarios for each configuration. Future research should explore hybrid systems that integrate DCMD and AGMD, optimizing thermal efficiency and promoting greater sustainability in industrial applications [42,43].

In light of the motivation of the present work, it is important to emphasize that the physical attributes of MD membranes, including the average pore size, thickness, and porosity, strongly impact the performance of membrane distillation (MD). These factors directly affect heat and mass transfer, which is essential for process efficiency. A critical factor that significantly impacts mass transfer mechanisms is pore size. In membranes with smaller pores, transport occurs predominantly by Knudsen diffusion. In this transport regime, the frequent interactions of vapor molecules with the pore walls enhance the selectivity and purity of the permeate since vapor transfer is effectively carried out without allowing liquid transport [44]. However, it is worth highlighting that membranes with inadequate pore sizes promote reduction in the driving force for mass transfer caused by the decrease in the vapor partial pressure gradient [45].

On the other hand, large pores favor convective transport, where the vapor flow is strongly influenced by the pressure gradient along the membrane. This increases permeability but augments the risk of liquid penetration into the pore, increasing permeate contamination and process failure [43,46]. In this sense, higher permeate fluxes are expected in membranes with larger average pore sizes, which in turn reduce the stability and selectivity of the process, increasing susceptibility to wetting [47,48].

Heat and mass transfer in the separation process via membrane distillation, as well as the energy efficiency of the system, are also influenced by the thickness of the membrane. Thicker membranes present greater thermal resistance, reducing the effect of heat loss by conduction through the membrane, the temperature polarization effect, preserving the temperature gradient necessary to promote the evaporation of water molecules close to the membrane surface [45]. In contrast, thicker membranes limit the mass transfer rate, presenting greater resistance to vapor transport, which can reduce the permeate flux [49]. In thinner membranes, thermal resistance is lower, increasing heat loss effects to the cold side of the membrane and decreasing the thermal efficiency of the process [50]. Although thin membranes are less efficient in terms of thermal resistance, they offer lower resistance to mass transfer, enabling higher permeate fluxes in the separation process [45]. Therefore, a fundamental characteristic of selective membranes for MD is that they must have sufficient thickness to minimize heat losses, without significantly compromising mass transfer across it. Therefore, there must be a balance between the thickness of the membrane and its effective thermal conductivity to maximize the efficiency of the separation process [45,49]. In terms of practical aspects, thicker membranes tend to be more resistant to mechanical damage; however, they require more thermal energy to maintain the temperature and vapor gradient. On the other hand, thinner membranes can be more fragile but offer higher productivity under optimized conditions [44,47].

Besides the thickness, another fundamental parameter in the performance of the membrane distillation (MD) process is porosity, defined as the ratio between the volume occupied by the pores and the total volume of the membrane. In mass transfer, larger porosities increase the effective mass transfer area, enhancing the evaporation rate and minimizing the resistance of the vapor flow. This improves the productivity of the separation process by increasing the permeate flow [45,47]. In heat transfer, previous studies show that increasing porosity by 15% can result in a 14.6% increase in thermal efficiency, offering a positive correlation between porosity and thermal efficiency [51]. Other important effects of larger porosity are the risk of wetting and the impact on mechanical strength. Membranes with very high porosity are vulnerable to wetting, since the existence of many interconnected pores facilitates the penetration of the liquid, compromising the separation and quality of the permeate [47,48]. High porosity also promotes structural fragility of the membrane, limiting durability and increasing the likelihood of mechanical damage during process operation. Thus, porosity must satisfy the compromise between operational efficiency and mechanical resistance to achieve long-term stability [44,48,51,52]. Therefore, conducting a comprehensive comparative analysis of membranes with different thicknesses, porosities, and pore diameters is critical to assess their suitability for specific operational contexts.

Another important aspect that determines the stability and performance of the long-term separation process is the hydrophobic properties of the membranes, characterized by physical and chemical properties that determine their resistance to contact with water and their ability to hinder pore wetting, where the contact angle, liquid inlet pressure (LEP), and the composition of the membrane material can be highlighted [53,54,55]. A measure of the hydrophobicity of a membrane is offered by the contact angle, which represents the angle formed at the contact of the membrane surface and the water droplet. Contact angles greater than 90° indicate that the surface of the material is hydrophobic, and for values above 150°, such a surface is considered superhydrophobic [53,54]. Less hydrophobic surfaces are more prone to partial or complete wetting of the pores, causing instability of the process in the long term, losing separation efficiency, and contaminating the permeate. A high contact angle favors efficient vapor transport and reduces the probability of a solution film forming in the pores, causing solutes (salts) to concentrate on the membrane surface, which can act as additional mass transfer resistance, reducing vapor flow [53,54,55]. Additionally, hydrophobic membranes with high contact angle values have a lower likelihood of particle and microorganism adhesion, contributing to the prevention of fouling and biofouling over time, which reduce the membrane’s service life and increase the frequency of maintenance, cleaning, or replacement [55].

Another parameter that defines the membrane’s ability to resist liquid penetration into the pores and that determines long-term performance is liquid inlet pressure (LEP). This parameter is defined as the minimum pressure required for the liquid to overcome the membrane’s surface tension and enter the pores. Therefore, if during the operation of the membrane distillation process the system exceeds the LEP value, pore wetting will occur, compromising the permeate quality [54]. In this sense, membranes that present higher LEP values are more resistant to wetting and often require fewer cleaning interventions, extending the membrane’s useful life. Membranes made of materials such as polytetrafluoroethylene (PTFE) and polyvinylidene fluoride (PVDF) are often selected because they present high LEP values due to the low surface energy that reduces interactions between the membrane surface and water molecules, contributing to greater hydrophobicity [55]. PTFE membranes are highly hydrophobic and chemically resistant, PVDF membranes are moderately hydrophobic and have good mechanical resistance, and polypropylene (PP) membranes have lower cost and reasonable hydrophobicity [56]. However, chemical and thermal stability of the process is essential to maintain hydrophobic properties, as well as monitoring and controlling the operating pressure to avoid exerting LEP, avoiding membrane wetting and increasing its useful life.

This study provides a detailed comparative analysis of three different commercial membranes made of polyvinylidene fluoride (PVDF), polytetrafluoroethylene (PTFE), and polyethylene (PE), employing two distinct MD modules, DCMD and AGMD. Experiments over a wide range of NaCl concentrations, from 7 to 70 g/L, in conjunction with theoretical modelling were utilized to assess the effects of key operational parameters—feed concentration, feed temperature, and flow rates—on permeate flux and thermal efficiency. The present analysis aims to verify the relative merits of the three different membrane materials, with their specific structural characteristics, operating on the DCMD and AGMD configurations.

## 2. Materials and Methods

### 2.1. Membranes and Module Configuration

Three different commercial polymeric membranes were used in the experiments, namely PVDF, PTFE, and PE. The membranes were characterized in terms of their structure, wettability, and thermal properties, as can be found in [5,57,58]. The detailed characteristics of each membrane, including mean pore size, porosity, and thickness, are provided in Table 1.

The membranes were tested in both the DCMD and AGMD configurations using flat-sheet modules designed and built for these setups. The DCMD module had an effective membrane area of 0.005062 m^2^, while the AGMD module had an effective membrane area of 0.006532 m^2^. The aim of our study is not to directly compare the performance of the present DCMD and AGMD configurations, since the modules are not in fact identical, but rather to analyze the impact of the membrane material and characteristics of three commercial membranes on each of these configurations. Nevertheless, so as to inspect the differences in flow pattern, we evaluated the hydrodynamic conditions on the feed side in both modules, which differ at most by 7%, by calculating the corresponding Reynolds number (Re). Previous studies indicate that, for similar Reynolds numbers, differences in module design do not significantly alter system performance, while the dominant transport mechanisms remain unchanged [59].

**Figure 1:**
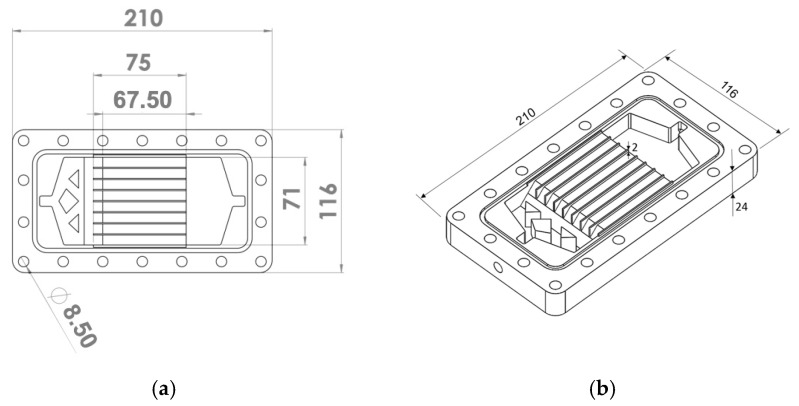
Drawings of the DCMD module in (**a**) top view and (**b**) geometric perspective (dimensions in mm).

**Figure 2:**
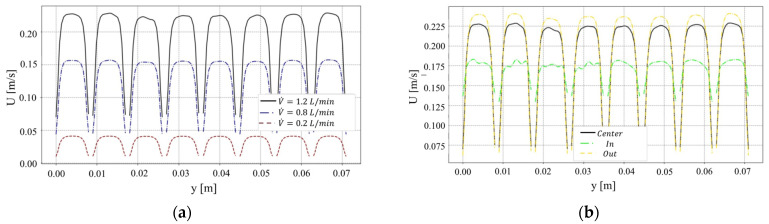
Simulated transversal velocity component profiles inside the module channels: (**a**) in the longitudinally central section for different volumetric flow rates, (**b**) in transversal sections near the inlet, at the center, and near the exit of the module for a volumetric flow rate of 1.2 L/min.

To design the geometry of the DCMD module with rectangular channels that ensure flow uniformity within the microchannels, CFD simulations were conducted, with the platform OpenFOAM (2021). These simulations varied the shape and characteristics of the inlet and outlet plenums on both the feed and distillate sides. For the analyzed range of volumetric flow rates, the chosen configuration with eight microchannels per side is shown in Figure 1. Velocity profiles obtained from the OpenFOAM simulations for various volumetric flow rates (0.2, 0.8, and 1.2 L/min) are shown in Figure 2. Figure 2a, at a central plane of the channels’ length, illustrates the uniformity of the velocity profiles at the various channels. Figure 2b provides velocity profiles at three different cross-sections (near the inlet, center, and outlet), reconfirming the fairly uniform distributions at different longitudinal positions.

**Figure 3:**
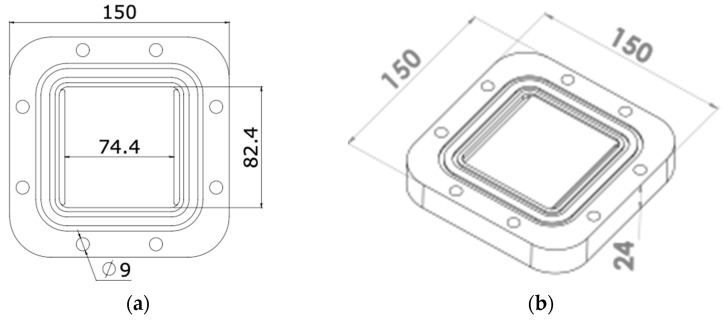
Drawings of the AGMD module in (**a**) top view and (**b**) geometric perspective (dimensions in mm).

The DCMD module’s flow uniformity for all analyzed flow rates supports the simplification assumption in the theoretical model towards formulating the heat and mass transfer process for a single representative channel. The AGMD module was designed as shown in Figure 3. It incorporates a 2 mm air gap between the membrane surface and the condensation plate.

In the case of the DCMD configuration, the design of the module is a critical factor. Non-uniform flow channels can generate stagnation zones, decreasing thermal efficiency and overall performance. Therefore, careful design is essential to ensure a more homogeneous distribution of heat flux and flow rates. On the other hand, in the AGMD configuration, the presence of the air gap reduces thermal variations and makes the system less sensitive to geometric variations. Janajreh et al. found that the air gap in AGMD can reduce thermal polarization by up to 38%, promoting more uniform heat distribution and greater process stability [25]. Furthermore, Bappy et al. demonstrated that the thickness of the air gap directly impacts performance: thinner gaps increase distillate flow and reduce thermal resistance, evidencing the stabilizing role of this component [60]. Finally, Liu et al. highlighted that air gap uniformity minimizes thermal losses, while DCMD remains more vulnerable to variations in channel design [61]. Thus, while DCMD requires an optimized design to avoid stagnation zones and maximize heat transfer, AGMD compensates for these limitations with the air gap, which stabilizes the process and maintains high efficiency even under less demanding design conditions.

### 2.2. Experimental Setup

**Figure 4:**
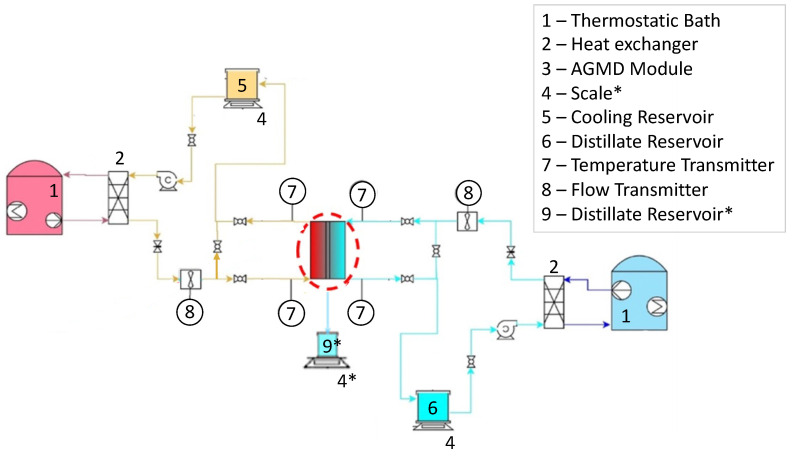
Schematic drawing of the benchtop membrane distillation system designed and built at LabMEMS/UFRJ—The elements marked with an asterisk (4* and 9*) are additional components (reservoir and scale) which are present only in the AGMD configuration.

A desalination bench was designed and built at LabMEMS/UFRJ for the evaluation of the distillation unit performance and characterization of commercial and modified superhydrophobic membranes, initially with a module in the DCMD configuration. The proposed experimental setup, shown in Figure 4, consists of two hydraulic circuits: the saline feed water circuit described by the orange lines and the distillate water flow circuit described by the blue lines.

In the DCMD configuration, the membrane is the only existing barrier that separates the feed and the permeate streams. The heating of the feed stream and the cooling of the permeate stream are carried out with the aid of two compact heat exchangers connected to two ultra-thermostatic baths to control the inlet temperatures of both streams. On the other hand, in the AGMD configuration, the salty water is heated and evaporated, while the cooling water is used to condense the evaporated water vapor. Here, the two water streams do not come into direct contact but are separated by an air gap, the condensing plate, and the membrane itself, thus preventing contamination of the cooling distilled water with salts or impurities in case of malfunction of the distillation process. The specification of operating parameters for this experimental setup are provided in Table 2.

The experimental setup includes temperature sensors (K-type thermocouples–chromel/alumel) at the inlet and outlet ports of the distillation module, four pressure sensors (Rucker, model RTP-420 0-0.6 bar, RüCKEN Instrumentos de Medição Ltda, São Paulo, Brazil), two microturbine volumetric flow rate meters (Badger BV1000-TRN-025-B, Badger Meter, Milwaukee, WI, USA), two probes for measuring feed and permeate electrolytic conductivity (Digimed DM32, Digimed, São Paulo, Brazil), and two fluid reservoirs, supported by precision scales (Marte AD 5002, Marte Científica, São Paulo, Brazil), where the instantaneous mass of the feed and permeate are measured and acquired to compute the mass flow rates. Unlike the DCMD configuration, which is simpler and uses a single circuit, the AGMD configuration includes additional components (elements marked with an asterisk, 4* and 9*), namely a third reservoir for collecting distilled water, where its quality is monitored by electrolytic conductivity, and its corresponding scale. These elements are not present in the DCMD configuration, which operates in a more simplified manner.

The utilization of NaCl concentrations exceeding 35 g/L in membrane distillation experiments is crucial for accurately simulating the conditions found in saline water sources, such as industrial and mining wastewater and reverse osmosis brines. Membrane distillation is a promising desalination technology capable of treating high-salinity waters, including petroleum reservoirs production water, while achieving high solute rejection rates [62,63].

## 3. Theoretical Model

The heat and mass phenomena that occur in the membrane distillation process are better understood through modeling and simulation to theoretically analyze the transport mechanisms and the interaction of parameters that influence the efficiency of the process. This task is essential to reduce costs and time in the experimental analysis for the various combinations of operational parameters, as well as to achieve proper design and operational optimization. In this sense, in addition to the influence of membrane characteristics, one of the main factors that affects the performance of the membrane distillation process is the temperature polarization phenomenon, evaluated by the temperature polarization coefficient (*TPC*) as

(1) $$TPC=\frac{T_{fm}-T_{pm}}{T_{f}-T_{p}}$$

Temperature polarization refers to the effect that the thermal boundary layer causes in reducing the temperature difference between the membrane surfaces ($T_{fm}$—temperature at the feed–membrane interface and $T_{pm}$—temperature at the membrane–permeate interface) with respect to the temperature difference between the fluid streams ($T_{f}$—average temperature of the fluid in the feed stream and $T_{p}$—average temperature of the fluid in the permeate stream). The mass transport of the volatile component (vapor) occurs through the pores of the membrane while heat is transferred both by the membrane solid matrix and by the vapor itself [59]. Also, the ratio between the total feed concentration and the concentration at the liquid–vapor interface on the feed side is called concentration polarization coefficient ($C_{fm}$—concentration at the feed–membrane interface and $C_{f}$—concentration at bulk feed solution). It can be determined from [64]

(2) $$CPC=\frac{C_{fm}}{C_{f}}$$

### 3.1. Heat Transfer

**Figure 5:**
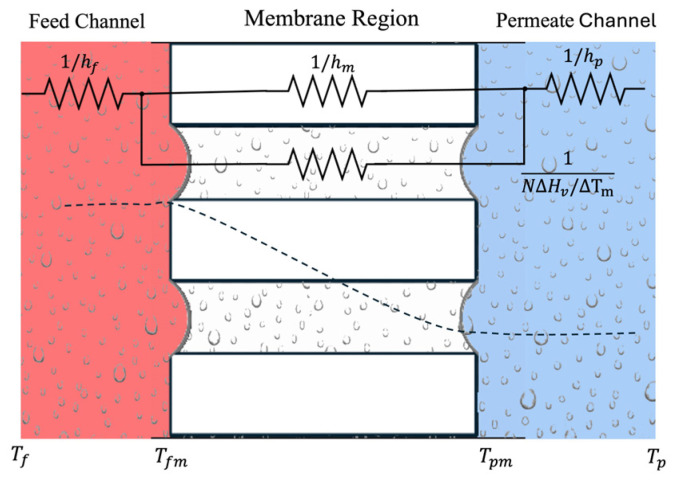
Schematic drawing of thermal resistances in a DCMD module.

From the feed channel to the permeate channel, the heat transfer process in a DCMD configuration is modelled across the three regions (feed stream, membrane, and permeate stream) as a lumped system, as shown in Figure 5: (i) the convective heat transfer $\left({\dot{Q}}_{con_{f}}\right)$ across the thermal layer of the feed stream in contact with the membrane, given by Equation (3), (ii) the transmembrane heat transport (${\dot{Q}}_{m}$) described by Equation (4), and (iii) heat transfer by convection $\left({\dot{Q}}_{conv\_p}\right)\;$across the thermal boundary layer of the permeate stream in contact with the other side of the membrane [59,65,66,67].

(3) $${\dot{Q}}_{{conv}_{f}}=h_{f}A_{m}(T_{f}-T_{fm})$$

(4) $$\dot{Q_{m}}={\dot{Q}}_{{vap}_{m}}+{\dot{Q}}_{cond\_m}=A_{m}\left[N_{w}\Delta H_{V}+\frac{k_{m}}{\delta_{m}}(T_{fm}-T_{pm})\right]$$

(5) $${\dot{Q}}_{conv\_p}=h_{p}A_{m}(T_{pm}-T_{p})$$

where $\dot{Q}$ is the heat transfer rate [W], h is the convective heat transfer coefficient [W/m^2^ K], $A_{m}$ is the area of the membrane normal to the heat flux [m^2^], $N_{w}$ is the permeate flux [kg/m^2^ s], $\Delta H_{V}$ is the latent heat of vaporization [J/kg], $k_{m}$ is the effective thermal conductivity of the membrane [W/m K], and $\delta_{m}$ is the nominal thickness of the membrane [m].

The effective conductivity $k_{m}$ can be estimated from the thermal conductivities of the solid and vapor phases $k_{s}$ and $k_{g}$ and the membrane porosity ε [-]. The parallel-resistance model (thermal conductivity in series) is the most used effective thermal conductivity model, followed by series-resistance (thermal conductivity in parallel) and the Maxwell I model [67,68,69,70]. In all these models, the fluid thermal conductivity in the bulk membrane $k_{mg}$, is made equal to the water vapor thermal conductivity, which is considered a temperature-dependent property. For the interface regions, however, the liquid water thermal conductivity is used. Here, the effective conductivity $k_{m}$ is described by the isostrain (parallel) model, Equation (6), which the literature indicates as adequate for membranes with solid and gas phases approximately parallel to each other [64,71,72,73].

(6) $$k_{m}=\left(1-\varepsilon \right)k_{s}+\varepsilon k_{g}$$

In the steady-state regime, Equations (3)–(5) can be associated to determine the surface temperatures at the membrane interfaces ($T_{fm}$ and $T_{pm}$) once the heat transfer coefficients are computed, equating the heat transfer rates in each region:

(7) $${\dot{Q}}_{{conv}_{f}}={\dot{Q}}_{m}={\dot{Q}}_{{conv}_{p}}$$

By means of the energy balance of Equation (7), and rewriting $k_{m}/\delta_{m}$ as $h_{m}$, the membrane surface temperatures on the feed ($T_{fm}$) and permeate ($T_{pm}$) sides can be obtained as

(8) $$T_{fm}=\frac{\left(h_{m}\left[T_{p}+\left(\frac{h_{f}}{h_{p}}\right)T_{f}\right]+h_{f}T_{f}-N_{w}\Delta H_{V}\right)}{h_{m}+h_{f}\left(1+\frac{h_{m}}{h_{p}}\right)}$$

(9) $$T_{pm}=\frac{\left(h_{m}\left[T_{f}+\left(\frac{h_{p}}{h_{f}}\right)T_{p}\right]+h_{p}T_{p}+N_{w}\Delta H_{V}\right)}{h_{m}+h_{p}\left(1+\frac{h_{m}}{h_{f}}\right)}$$

The present geometric and operational conditions warrant laminar flow regime on both fluid streams, so the following correlation for Nusselt number, previously employed for the DCMD configuration [71,73], is adopted:

(10) $$Nu\equiv \frac{h\;d_{h}}{k}=4.36+\frac{0.036\;Re\;Pr\;\left(\frac{d_{h}}{L}\right)}{1+0.0011{\left[Re\;Pr\;\left(\frac{d_{h}}{L}\right)\right]}^{2/3}},\;\;\;\;Re<2100$$

where *k* is the fluid thermal conductivity [W/m K], *d_h_* is the hydraulic diameter of the channel, *Re* is the Reynolds number, Pr is the Prandtl number, and L is the channel length.

### 3.2. Mass Transfer

The permeate flux through the membrane ($N_{w}$) is described by the Dusty-–Gas model, based on the Maxwell–Stefan relationships. Neglecting surface diffusion, the Dusty–Gas model is written as [72]

(11) $$\frac{J_{w}}{D_{w}^{k}}+\frac{p_{a}J_{w}-p_{w}J_{a}}{D_{wa}^{o}}=-\frac{1}{RT_{m}}𝛻p_{w}$$

where $J_{w}$ and $J_{a}$ are the molar fluxes of water vapor and air [mol/m^2^ s], $p_{w}$ and $p_{a}$ are the partial pressures of water vapor and air inside the pore [Pa], $D_{w}^{k}$ is the Knudsen diffusivity, $D_{wa}^{o}$ is the molecular diffusivity of water in the air, $T_{m}$ is the thermodynamic average temperature inside the pores, and R is the universal gas constant.

Therefore, the distillate flux, $N_{w}$, is determined from the permeate molar flux, $J_{w}$, obtained from Equation (11) neglecting viscous transport and surface diffusion, zero air flux ($J_{a}≅0$) inside the pores, and considering Dalton’s law [74,75]:

(12) $$N_{w}=J_{w}M_{w}$$

(13) $$J_{w}=C_{hp}\frac{D_{wa}^{o}}{R\;T_{m\;}\delta_{appa}}\ln\left(\frac{D_{wa}^{o}-D_{eff}{\;p}_{f}^{vap}}{D_{wa}^{o}-D_{eff}{\;p}_{p}^{vap}}\right)$$

where $\delta_{appa}$ is the membrane apparent thickness, $M_{w}$ is the molecular mass of water, $D_{eff}$ is the effective diffusion coefficient, and $p_{f}^{vap}$ and $p_{p}^{vap}$ are the partial vapor pressures at the membrane interface on the feed and permeate sides, respectively.

Considering that the phenomenon of capillary depression is a consequence of the hydrophobicity of the membrane, refs. [76,77] adopted the two correction factors for the distillate mass flux, namely $C_{hp}$ and $\delta_{appa}$. The curved region formed at the entrance of the membrane pores increases the total area of the liquid–vapor interface according to the hydrophobicity degree (related to the contact angle, *θ*), which varies for each membrane. This effect is accounted for by factor $C_{hp}$. Additionally, due to this increase on the interfacial surface, the distance between the two liquid–vapor interfaces is reduced; thus, an apparent membrane thickness ($\delta_{appa}$) was also employed in the mass flux relation [78]:

(14) $$C_{hp}=\frac{2}{\left[1+\sin\left(\theta \right)\right]}$$

(15) $$\delta_{appa}=\delta -(\delta_{{interf}_{feed}}+\delta_{{interf}_{perm}})$$

The interface thickness ($\delta_{{int}_{feed}}$) for the feed–membrane interface and $\delta_{{int}_{perm}}$ for the membrane–distillate interface are evaluated as the lengths of the portion of the pore volume that is occupied by the liquid at the pore channel entrance, which is approximated as the volume of a spherical cap, being a function of the mean pore diameter, *dp*, and the contact angle, *θ* [78]:

(16) $$\delta_{int}=\frac{d_{p}}{2cos(\theta )}\left[\sin\left(\theta \right)-1\right]$$

The vapor partial pressure at the membrane interface, $p_{i}^{vap}$, is a function of the temperature estimated according to the Antoine equation [72]:

(17) $$p_{i}^{vap}=exp\left(23.1964-\frac{3816.44}{T_{m,i}-46.13}\right)$$

where $T_{m,i}$ is the fluid–membrane interface temperature in [K]. For the feed side, the saline concentration (NaCl) must be considered, since the saline concentration reduces the partial pressure. For non-ideal binary mixtures, the vapor partial pressure is often described as

(18) $$p_{i}^{vap\prime }=x_{w}\;a_{w}\;p_{i}^{vap}$$

where $x_{w}$ is the water molar fraction, $p_{i}^{vap}$ is the water vapor partial pressure given by the Antoine equation, Equation (17), and $a_{w}$ is the activity coeficient, related to the feed sodium chloride molar fraction $X_{NaCl}$ as

(19) $$a_{w}=1-0.5X_{NaCl}-10X_{NaCl}^{2}$$

Finally, the effective diffusion coefficient is expressed as function of the Knudsen diffusivity $D_{w}^{k}$, and the molecular diffusivity of water in air, $D_{wa}^{o}$, besides the total pressure inside the pores, *P*:

(20) $$D_{eff}=\left(\frac{D_{w}^{k}D_{wa}^{o}}{D_{wa}^{o}+PD_{w}^{k}}\right)$$

(21) $$D_{w}^{k}=\frac{\varepsilon d_{p}}{3\tau }\sqrt{\frac{8RT_{m}}{\pi M_{w}}}N_{w}=J_{w}M_{w}$$

(22) $$D_{wa}^{o}=4.46\cdot 10^{-6}\frac{\varepsilon }{\tau }T_{m}^{2.334}$$

where *ε* is the membrane porosity, *τ* is the membrane tortuosity [79], and $d_{p}$ is the membrane pore diameter.

Several tortuosity models can be found in the literature [80,81,82,83,84]. A critical review of some tortuosity models was performed in [85]. Recently, to better understand the effects of the tortuosity on the distillate mass flux in DCMD systems and to better evaluate their adequacy in describing this output parameter, [86] evaluated eleven tortuosity models (eight based on geometrical hypothesis and three based on fractal theory), comparing their predictions with experimental results. The geometry-based tortuosity models are supported by distribution of Euclidean distances between pores of the membrane structure. On the other hand, fractal theory-based porous media assume that molecules diffuse along a path within a fractal system. Nevertheless, in both Euclidean and fractal models, tortuosity is always estimated from relationships in terms of membrane porosity. In the present work, for the morphology of the porous membranes used, we use the expression proposed in [87,88,89]:

(23) $$\tau =\frac{{\left(2-\varepsilon \right)}^{2}}{\varepsilon }$$

For AGMD, the distillate mass flux can be calculated by compensating for the resistance of the air space, as follows [90]:

(24) $$J_{w}=\frac{{(P}_{wa}-P_{c,f})}{R_{ag}}\;$$

where

(25) $$R_{ag}={\left[\frac{\varepsilon DP}{\delta_{a}P_{ag,log}R}\frac{M_{v}}{\left(T_{ag,avg}+273.15\right)}\;\right]}^{-1}$$

where *P_c,f_* is the vapor pressure at the air gap interface of the condensing plate, *R_ag_* is the air gap resistance, *δ_a_* is the air gap width, *P_ag,log_* is the log mean pressure in the air gap, and *T_ag,log_* is the log mean temperature in the air gap. The total resistance in the AGMD model can then be calculated by

(26) $$R_{AGMD}=R_{m}+R_{ag}$$

Then,

(27) $$R_{AGMD}=\left(\frac{\tau \delta }{\varepsilon }+\delta_{ag}\right)\frac{P_{ag,log}}{DP_{t}}\frac{R_{g}T_{ag,avg}}{M_{v}}+{\left[\frac{2\varepsilon r}{\tau \delta }{\left(\frac{8M_{v}}{\pi R_{g}T_{m}}\right)}^{0.5}\right]}^{-1}$$

where *δ*, *δ_ag_*, *ε*, *τ*, and *r* are the thickness of the membrane, the thickness of the air gap, the porosity of the membrane, the tortuosity of the pores, and the average pore size of the membrane, respectively. *D* is the diffusion coefficient of water vapor through the air gap, *P_t_* is the total air and water vapor pressure.

### 3.3. Implementation of the Computational Model

The heat and mass transfer model of the DCMD system, given by Equations (1)–(23), is evaluated through a computational program developed and implemented in the Matlab–Simulink platform (R2019a) for the theoretical prediction of the distillate mass flux, besides other variables. Since there is an interdependency between distillate mass flux and the temperatures at the membrane–fluid interfaces, i.e., $N_{w}=f\left(T_{fm},\;T_{pm}\right)$, $T_{fm}=f\left(N_{w}\right),$ *and* $T_{pm}=f\left(N_{w}\right)$, it is necessary to employ an iterative procedure for the simultaneous convergence of all dependent variables. Initially, all morphological membrane parameters must be defined, including physical properties, module geometry, and process operational conditions. The thermophysical properties of water (density, specific heat, thermal conductivity, and viscosity) are evaluated according to the database of the EES software v.9.9 (Engineering Equation Solver). The physical and chemical properties for the saline solutions are evaluated according to the correlations presented in [91]. After estimating the temperatures at the interfaces of the membrane and determining the heat and mass transfer parameters, the convergence for the distillate mass flux is verified, in each iterative step, to within a predefined relative error criterion, $tol=10^{-6}$.

Two performance metrics are used to evaluate the energy efficiency in each experiment. The parameters are the gain output ratio (*GOR*) and the specific energy consumption (*SEC*). *GOR* is defined as the ratio of the heat of the water vapor to the total input heat, which remains equal to or less than one in cases when latent heat is not recovered [50], and it is given by

(28) $$GOR=\frac{{\dot{m}}_{perm}\Delta H_{v}}{{\dot{Q}}_{in}}$$

where ${\dot{m}}_{perm}=J_{w}WL$; *W*, *L* are the channel width and length, $\Delta H_{v}$ is evaporation latent heat, ${\dot{Q}}_{in}$ is the heat transfer rate.

Another essential parameter for such comparisons is the specific energy consumption (*SEC*), which assesses the energy efficiency of the process. The *SEC* is an indicator for evaluating the relationship between the energy consumed and the result obtained in the membrane distillation process [92]. This parameter can be calculated as

(29) $$SEC=\frac{{\dot{Q}}_{in}\rho_{w}}{J_{w}A}$$

where $\rho_{w}$ is water density and *A* is the effective area of the membrane.

## 4. Results and Discussion

This section presents the results of the laboratory bench experiments with the three commercial membranes tested in the two designed modules of DCMD and AGMD configurations. The effects of the membrane materials and parameters and their impacts on the membrane distillation process are described and discussed. The performance characteristics of the module configurations employed in the MD process are also presented and analyzed. Finally, comparisons between the bench-scale experimental results and those of the implemented theoretical model are provided, validating the results of the developed computational simulation.

**Figure 6:**
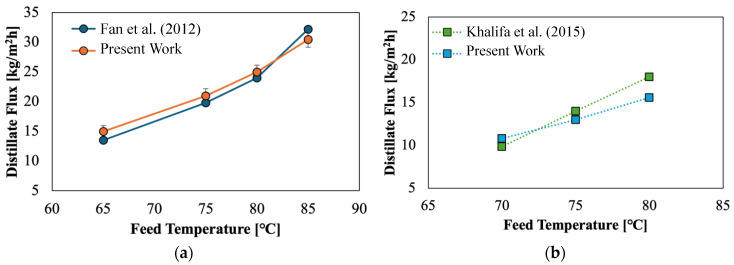
Comparison of distillate mass fluxes as a function of feed temperature in (**a**) DCMD with PVDF membrane (porosity of 78%, average pore radius of 0.49 μm, and thickness of 82 μm) [93] and (**b**) AGMD with PTFE membrane (porosity of 80%, pore size of 450 nm, and thickness of 153.9 μm) [94] between this study and the literature data for the same membrane material.

First, we verified that the obtained permeate fluxes are comparable with the literature results for both configurations, considering the same membrane materials and feed temperatures [93,94]. A direct comparison between these sets of results is not the aim here due to the differences pointed out above, but Figure 6 shows that the present experimental results are consistent with similar runs in the literature.

### 4.1. Analysis of Membrane Parameters in the MD Performance

**Figure 7:**
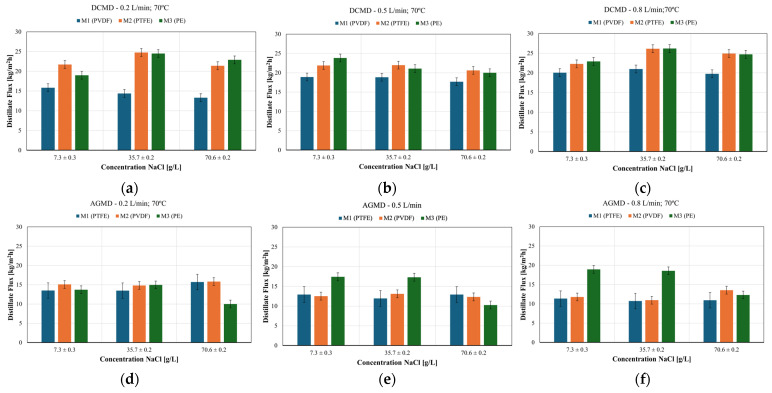
Distillate fluxes for feed solutions at different feed flow rates for (**a**) DCMD at 0.2 L/min; (**b**) DCMD at 0.5 L/min; (**c**) DCMD at 0.8 L/min; (**d**) AGMD at 0.2 L/min; (**e**) AGMD at 0.5 L/min; (**f**) AGMD at 0.8 L/min.

Figure 7 shows the distillate flux under the same operational conditions for flow rates of 0.2 L/min, 0.5 L/min, and 0.8 L/min in the DCMD (a, b and c) and AGMD (d, e and f) modules for all NaCl concentrations used in this experiment. The general trend observed is a reduction in the distillate flux with increasing NaCl concentration and feed flow rate. This occurs due to the increase in the concentration polarization effect, which reduces the vapor pressure difference across the membrane, impairing the mass transport efficiency. As expected, the distillate fluxes were higher in the experiments using the M3 membrane. As shown in Table 1, the PE membrane has the smallest thickness (110 µm), the largest pore diameter (0.32 µm), and the highest porosity (85%). These characteristics reduce mass transfer resistance, thereby increasing vapor flux compared to the other two samples. However, these same characteristics make the membrane more susceptible to the wetting effect in solutions with high salt concentration. This increases permeability but also increases the risk of liquid penetration into the pores, leading to higher permeate contamination and potential process failure. In references [95,96], it is argued that high porosity (>75%) increases vapor flux but requires careful management to avoid excessive thermal losses. They also emphasize that the combination of such parameters can compromise the structural integrity of the membrane, making it more susceptible to mechanical damage, pore intrusion, and wetting.

Overall, for low concentrations (7 g/L and 35 g/L), the M3 membrane showed better performance, especially in the AGMD module. For higher concentrations (70 g/L), M1 and M2 were expected to offer greater resistance to wetting and improved stability. The AGMD configuration is more effective in reducing the effects of thermal polarization and wetting, favoring the use of higher thermal conductivity membranes, such as M3, especially for low concentrations.

### 4.2. Comparison of Energy Efficiency

**Figure 8:**
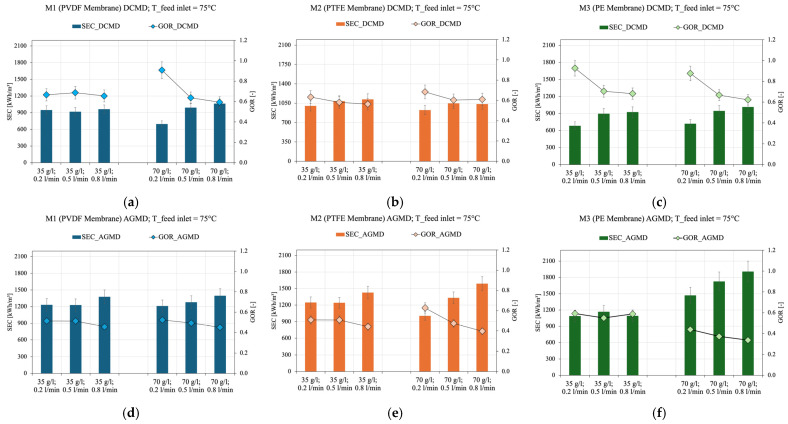
Energy efficiency parameters SEC and GOR as a function of feed temperature in DCMD (**a**–**c**) and AGMD (**d**–**f**) configurations for three different commercial membranes.

Figure 8 presents information on the energy performance of the three commercial membranes in the two different modules here analyzed, using the Gain Output Ratio (*GOR*) and Specific Energy Consumption (*SEC*) indicators at different salt concentrations in the feed solution (35 g/L and 70 g/L) and different feed flow rates, maintaining a feed inlet temperature of 75 °C.

An increase in the feed flow rate increases the distillate flux, as previously discussed. However, Figure 8 shows a trend of increasing specific energy consumption with increasing feed flow rate. This effect can be associated with a reduction in residence time in the feed current. As the flow rate increases, both the heat transfer efficiency and the evaporation rate can be affected. Consequently, energy consumption may increase without a proportional gain in distillate production. Heat losses to the ambient may also be affected, reducing the energy efficiency of the process, but this effect was not quantified here.

It should be noted that for the M3 (PE) membrane, increasing the salt concentration, more noticeably reduces the energy efficiency of the process, an effect that is more pronounced in the AGMD configuration due to concentration polarization, which reduces the vapor pressure gradient [97]. On the other hand, for M1 (PVDF) and M2 (PTFE) membranes, no significant effects due to increase in the salt concentration have been observed on the energy efficiency indicators. This behavior can be attributed to the high hydrophobicity of these two membranes, which have larger contact angles (>120°) compared to the M3 (PE) membrane (<100°). The literature corroborates this observation, highlighting that the M1 (PVDF) and M2 (PTFE) materials in general present better performance due to their high hydrophobicity and resistance to wetting, especially in high-salinity conditions [3]. The high hydrophobicity of the membrane prevents liquid water from penetrating the pores, ensuring that transport occurs only in the vapor phase. The addition of salts reduces the surface tension of the feed solution, which can increase the water layer across the membrane, promoting the formation of liquid films on the membrane surface. In less hydrophobic membranes, the occurrence of partial or total wetting is more likely, since small amounts of solution can enter the pores, reducing the efficiency of vapor transport, leading to an increase in specific energy consumption (*SEC*) and a decrease in thermal efficiency (*GOR*) [97,98,99]. A higher NaCl concentration lowers the surface tension of the feed solution, making it easier for the liquid to penetrate the membrane pores. This can compromise vapor transport and increase specific energy consumption (*SEC*), in addition to reducing the process thermal efficiency (*GOR*), as seen in Figure 8c,f.

Thus, it is concluded that the M3 (PE) membrane is the least suitable for low- and medium-salinity solutions, regardless of the module configuration used. However, at high salinities, the energy efficiency of M3 (PE) decreases, especially in the AGMD configuration. In contrast, the M1 (PVDF) membrane offers a versatile solution, as it maintains the energy efficiency of the process over a wide range of salinity levels. Finally, for applications involving high-salinity solutions, M2 (PTFE) demonstrates superior energy performance, especially in the AGMD configuration.

### 4.3. Temperature Polarization

**Figure 9:**
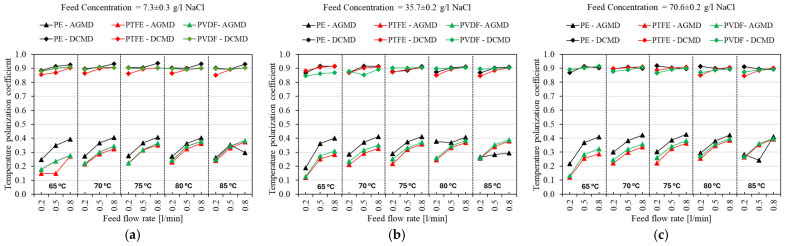
Temperature polarization coefficient (*TPC*) as a function of feed flow rate and feed inlet temperature for three different commercial membranes (PE, PTFE, and PVDF) in the AGMD and DCMD modules. (**a**) 7 g/NaCl; (**b**) 35 g/NaCl; (**c**) 70 g/NaCl.

Figure 9 presents the temperature polarization coefficient (*TPC*) as a function of feed flow rate and temperature for the three different commercial membranes (M1, M2, and M3) in the AGMD and DCMD configurations, using saline feed solutions with NaCl concentrations of 7 g/L, 35 g/L, and 70 g/L, as shown in Figure 9a–c, respectively. In general, the *TPC* increases with the rise in feed flow rate (from 0.2 to 0.8 L/min) for all temperatures and membrane materials, regardless of concentration and process configuration. The increase in flow rate decreases the thickness of the thermal boundary layer near the membrane surface, improving heat transfer and reducing temperature polarization, which enhances thermal efficiency and *TPC* values [100,101,102].

A moderate effect of feed temperature (ranging from 65 °C to 85 °C) is also observed for all NaCl concentrations, particularly in the AGMD configuration. Although an increase in feed temperature intensifies the vapor pressure gradient and distillate flux, it simultaneously amplifies the polarization effect, limiting the effective thermal gradient across the membrane [100,102,103]. This phenomenon is evidenced in Figure 9, where the *TPC* values appear to reach a saturation point in the results for M3, which is made of the material with the highest thermal conductivity and the lowest thickness. The effect of feed temperature is significant at lower temperatures (65–75 °C), particularly M1 and M2, membranes with lower thermal conductivity, and greater thickness. In this case, the thermal gradient is not yet as intense, allowing the *TPC* to increase significantly within this range. Thus, the system can better harness the temperature increase to enhance vapor pressure. However, beyond 75 °C, the rise in *TPC* is weakened, and consequently, the positive effect on vapor pressure difference is diminished, leading to more significant heat losses [100,102,103].

### 4.4. Concentration Polarization and Salt Rejection

**Figure 10:**
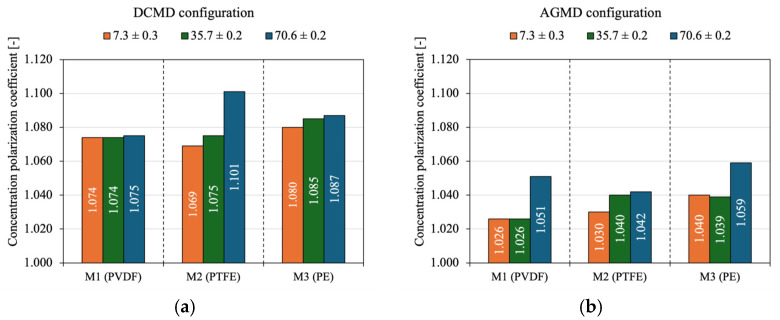
Effect of feed salinity on the concentration polarization coefficient (*CPC*) for the three commercial membranes, considering V_feed_ = 0.5 L/min and T_feed_ = 80 °C in (**a**) DCMD module; (**b**) AGMD module.

Figure 10 evaluates the effect of feed solution salinity on the concentration polarization of the three commercial membranes in the DCMD module (Figure 10a) and the AGMD module (Figure 10b), considering a feed flow rate of 0.5 [L/min] and a feed temperature of 80 [°C]. *CPC* values equal to one indicate no concentration polarization, meaning that the membrane interface concentration is the same as that of the bulk solution. *CPC* > 1 indicates the occurrence of concentration polarization, while *CPC* > 2 indicates severe solute accumulation, which can significantly impact the distillate flux and promote fouling and scaling effects on the membrane [64,104,105,106].

There is a trend of increasing *CPC* as the NaCl concentration in the feed solution increases. M1 generally showed the lowest *CPC* values, with an average of 1.074 for DCMD and 1.034 for AGMD. The membrane, with moderate hydrophobicity (128.2°), porosity (75%), and smaller pores (0.22 µm), offers better resistance to concentration polarization in medium- and high-salinity applications, as it balances vapor permeability and solute accumulation control [107,108]. It is noteworthy that the highest observed *CPC* value was 1.101 at a feed concentration of 70 g/L for M2, indicating that the concentration at the membrane’s hot surface was approximately 10.1% higher than that of the bulk feed stream. In highly hydrophobic membranes such as PTFE (143.4°), a water-repellent surface creates a stable solid–liquid interface essential for vapor transport. When water evaporates, the liquid volume near the interface decreases rapidly. Since Na⁺ and Cl^−^ ions do not evaporate, they tend to concentrate quickly, forming a concentrated solute layer. Therefore, if the feed flow velocity is low, there is a higher tendency for local solute accumulation, intensifying concentration polarization effects [104,105,108]. For M3, feed salinity had a minimal effect on concentration polarization. The low hydrophobicity of the PE membrane (contact angle of 99.5°) tends to increase the presence of water at the interface, which can temporarily dilute the solute layer, minimizing the effect of salinity on concentration polarization, especially at low concentrations as seen in the AGMD module (Figure 10b).

**Figure 11:**
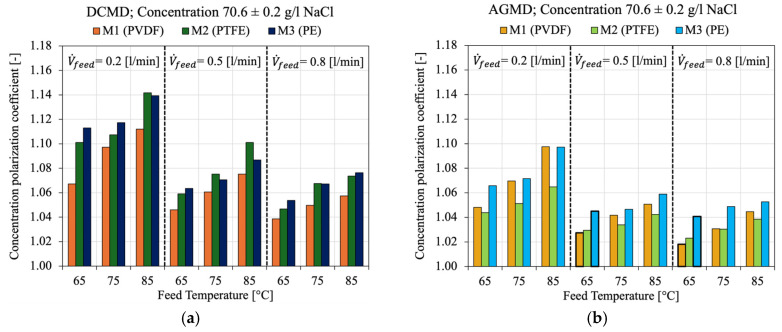
Effect of feed flow rate and feed temperature on the concentration polarization coefficient (*CPC*), considering 70 g/L of NaCl, for the three commercial membranes. (**a**) DCMD module; (**b**) AGMD module.

Figure 11 presents the behavior of the *CPC* under different feed flow rates (0.2, 0.5, and 0.8 L/min) and feed temperatures (65, 75, and 85 °C) for 70 g/L NaCl for the three commercial membranes M1, M2 and M3 in the DCMD (Figure 11a) and AGMD (Figure 11b) modules. As the feed velocity increases from 0.2 L/min to 0.8 L/min, the *CPC* consistently decreases for all membranes and temperatures. Particularly, for M2 (PTFE) in DCMD at 85 °C, the *CPC* value is 1.14 at a flow rate of 0.2 L/min and decreases to 1.07 at 0.8 L/min. This indicates that at the lower flow rate (0.2 L/min), the concentration near the membrane surface was 14% higher than that of the bulk, and it was reduced to 7% when the flow rate increased to 0.8 L/min. Therefore, increasing the flow rate led to a reduction of approximately 50% in the effects of concentration polarization. This trend was also observed in M1 and M2. This occurs because higher velocities promote faster solute boundary layer renewal, further dispersing the accumulated ions. Thus, increasing the flow rate reduces the thickness of the diffusive boundary layer, decreasing solute accumulation at the membrane interface and minimizing the effects of concentration polarization [104,105,106]. The temperature effect on *CPC* can also be observed, *CPC* increases with rising temperature for all feed flow rates and membrane types. Higher temperatures enhance the water evaporation rate, intensifying solvent removal and concentrating solutes at the membrane interface, thereby augmenting concentration polarization, especially in membranes with high porosity (M2 and M3) [104,107,108].

**Figure 12:**
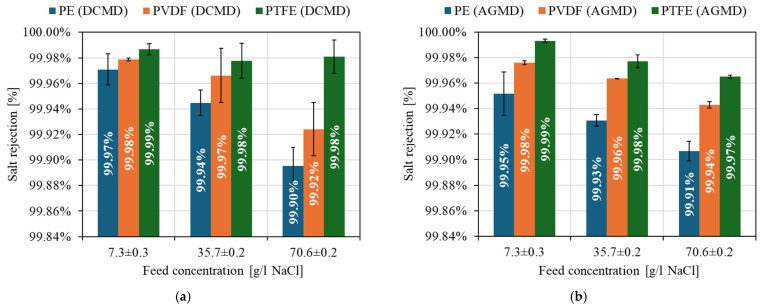
Salt rejection (%) for the different membranes analyzed at different feed salinity concentrations. (**a**) DCMD; (**b**) AGMD.

Finally, Figure 12 shows the salt rejection rate for the three commercial membranes (M1, M2 and M3) under the different feed concentrations and for the two modules implemented: DCMD (Figure 12a) and AGMD (Figure 12b). For all membranes, salt rejection surpassed 99.90%. This demonstrates efficient performance in retaining saline ions (Na⁺ and Cl^−^) while allowing water vapor to pass through. The M2 membrane exhibited the highest salt rejection (>99.97% under all conditions), followed by the M1 and M3 membranes. The high hydrophobicity of the PTFE membrane (143.4°) and its smaller pore diameter (0.22 µm) ensure selective vapor transport while blocking ion passage [104,105,107]. An effect of increasing feed concentration on the reduction in salt rejection for all membranes, regardless of the module configuration used, was also observed. The increase in feed salinity intensifies osmotic pressure and concentration polarization effects, resulting in reduced efficiency of selective vapor transport [104,105,108]. In this context, for high-salinity conditions, membranes with small pores (0.22 µm) and moderate to high hydrophobicity, such as M1 or M3, are recommended to maximize the quality of the distilled water produced in the separation process.

### 4.5. Comparison of Experimental Results and the Mathematical Models

**Figure 13:**
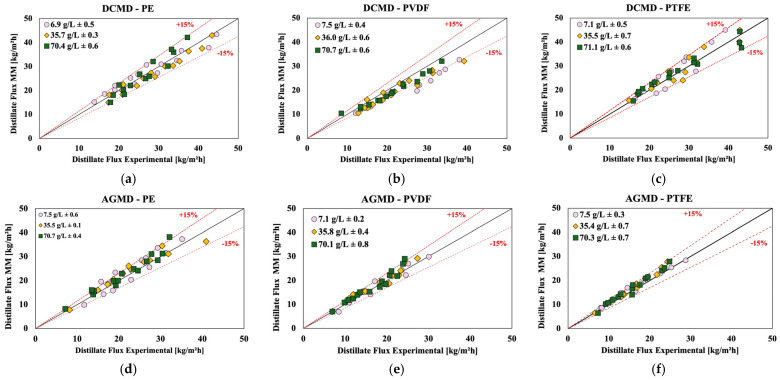
Comparison between experimental permeate fluxes and those predicted by the mathematical models for the DCMD and AGMD configurations with different membranes (PE, PVDF and PTFE) at NaCl concentrations of 7, 35 and 70 g/L and flow rates of 0.2, 0.5 and 0.8 L/min: (**a**) DCMD–PE; (**b**) DCMD–PVDF; (**c**) DCMD–PTFE; (**d**) AGMD–PE; (**e**) AGMD–PVDF (**f**) AGMD–PTFE.

Figure 13 compares results of the permeate fluxes obtained experimentally and predicted by the mathematical models previously described for the DCMD and AGMD membrane distillation configurations. Each graph represents a specific combination of configuration (DCMD and AGMD) and membrane material (PE, PVDF and PTFE), and shows the relation between the experimental values (on the x-axis) and the predicted values (on the y-axis) for different NaCl concentrations (7, 35 and 70 g/L) and different flow rates (0.2, 0.5 and 0.8 L/min). The figures include reference lines for 15% relative deviation, which allows to assess the accuracy of the mathematical model in role to the experimental data. In all graphs, most of the points are concentrated close to the line of equality (y = x), indicating that the mathematical model presents good agreement with the experimental data for both configurations and for all membranes analyzed. The great majority of points lie within the bounds of the ±15% deviation lines, indicating that the model is a reliable tool in predicting MD performance under a variety of operating conditions. Some slight discrepancies can be noted, especially for the PVDF membrane in the DCMD configuration, where a few points exceed the 15% deviation bounds.

## 5. Conclusions

The study presented a comprehensive comparative analysis of three commercial hydrophobic membranes of different materials (PE, PVDF, and PTFE) for two distinct modules representing the Direct Contact Membrane Distillation (DCMD) and Air Gap Membrane Distillation (AGMD) configurations. In general, for the present set of experiments and having in mind the differences between the two modules, the DCMD configuration exhibited higher distillate fluxes, while the AGMD configuration demonstrated superior thermal performance and stability under higher-salinity conditions. The PE membrane achieved higher distillate flux under low-salinity conditions due to its high porosity (85%), larger pore diameter (0.32 µm), and lower thickness (110 µm). However, it showed a decline in performance at high salt concentrations (70 g/L). The performance of the PTFE membrane under high salinity was superior to that of PVDF due to its higher porosity (85%) and greater contact angle (143.4°). Therefore, membranes with high porosity and large pore diameter are natural candidates towards maximizing distillate flux, although they require greater attention regarding wetting and thermal losses. Increasing the feed stream temperature significantly improved the distillate fluxes in both modules here evaluated. This effect was more significant in the AGMD configuration due to the reduced heat losses through the membrane. Due to their higher hydrophobicity, the PTFE membranes exhibited superior stability and separation quality, especially under high-salinity conditions. The experimental data correlated well with the proposed theoretical models, validating the simulation results and distillate flux predictions, offering a valuable tool for future efforts of simultaneously optimizing membrane and operational parameters.

These findings underscore the critical balance between maximizing water flux and minimizing energy consumption, offering valuable insights for selecting or manufacturing membranes towards optimal properties and operational configurations to the specific demands of each application. It is also important to emphasize that membrane distillation is a promising and adaptable technology for desalination and wastewater treatment in different sectors, especially in integrating and using low-exergy waste heat.

## Figures and Tables

**Table 1:** Physical properties of hydrophobic membranes.

	Units	M1 (Millipore)	M2 (Millipore)	M3 (Aquastill)
**Membrane Material**	-	Polyvinylidene fluoride	Polytetrafluoroethylene	Polyethylene
**Support layer material**	-	-	High-Density polyethylene (HDPE)	-
**Membrane Thickness**	μm	125	150	110
**Average Pore Size**	μm	0.22	0.22	0.32
**Porosity (** ε **)**	%	75	85	85
**Water Contact Angle (** θ **)**	°	128.2° (±2%)	143.4 (±2%)	99.5° (±2%)
**Water Entry Pressure**	bar	8.09	3.68	2.38
**Thermal conductivity ^1^**	W/m·K	0.19 to 0.25	0.23 to 0.25	0.33 to 0.35

^1^ Thermal conductivity of the solid matrix of the membrane.

**Table 2:** Operating parameters for the experimental desalination setup.

Component	Unit	Values
Feed Tank	L	5
Permeate Tank	L	5
Feed Temperature	°C	65; 70; 75; 80; 85
Permeate Temperature	°C	32.5
Feed flow rate	L/min	0.2; 0.5; 0.8
Permeate flow rate	L/min	0.5
Feed Concentrate	g/L	7; 35; 70

## Data Availability

The data presented in this study are available on request from the first author due to sponsorship restriction.
